# Gastrointestinal Stromal Tumour of the Appendix: A Very Rare Entity

**DOI:** 10.7759/cureus.59780

**Published:** 2024-05-07

**Authors:** Jacob D Williams, Chia Y Kong, Rudi Schmigylski, Krsty Nale, Muhammad Mirza

**Affiliations:** 1 Department of General Surgery, Dumfries and Galloway Royal Infirmary, Dumfries, GBR; 2 Academic Unit of Surgery, University of Glasgow, Glasgow, GBR; 3 Department of Pathology, Dumfries and Galloway Royal Infirmary, Dumfries, GBR

**Keywords:** appendix tumours, gist risk, appendix neoplasms, gist, gastrointestinal stromal tumour

## Abstract

A 74-year-old man who presented with upper abdominal pain was found to have an incidental appendiceal mass on cross-sectional imaging. He underwent a laparoscopic appendicectomy with histopathological examination confirming a completely resected appendiceal gastrointestinal stromal tumour (GIST). Appendiceal GISTs are rare. Therefore, there is limited evidence to guide risk stratification and management with extrapolation of prognosis from data on GISTs at other sites. This paper highlights the rarity of these tumours and presents another case which correlates well with the existing but limited literature. There is a need to maintain a registry of this rare disease entity with the maintenance of longer-term follow-up data.

## Introduction

Gastrointestinal stromal tumours (GISTs) are rare tumours derived from the intestinal cells of Cajal and their precursor neural crest cells, accounting for fewer than 1% of gastrointestinal tumours [1].

These lesions can develop throughout the entire gastrointestinal tract, with the stomach being the most common site followed by the small bowel [2]. Thereafter sites of GIST include the colon and rectum [2]. The appendix is an exceptionally rare site, accounting for 0.1-0.2% of all GISTs [3].

GISTs at different anatomic sites carry significantly different risks of metastasis. For example, the risk of metastasis is five times greater for intestinal GISTs when compared with gastric GISTs of a similar size and mitotic profile [4].

Despite evidence of a spectrum of biological behaviours of GISTs dictated by the anatomical site of origin, the prognosis and risk for appendiceal GISTs continue to be inferred and extrapolated from GISTs at other sites [2,5].

## Case presentation

Case history 

A 74-year-old man presented to our unit, a relatively rural district general hospital, with a one-day history of severe upper abdominal pain. He had a significant past medical history of chronic obstructive pulmonary disease, polycystic kidney disease, gout, vitamin B12 deficiency, and a hiatus hernia. He also had a significant surgical history of a previous open inguinal hernia repair and a previous open repair of a ruptured abdominal aortic aneurysm.

He had a normal white cell count but a mildly raised C-reactive protein of 22 mg/dL. His liver function tests and serum amylase were normal.

**Figure 1:**
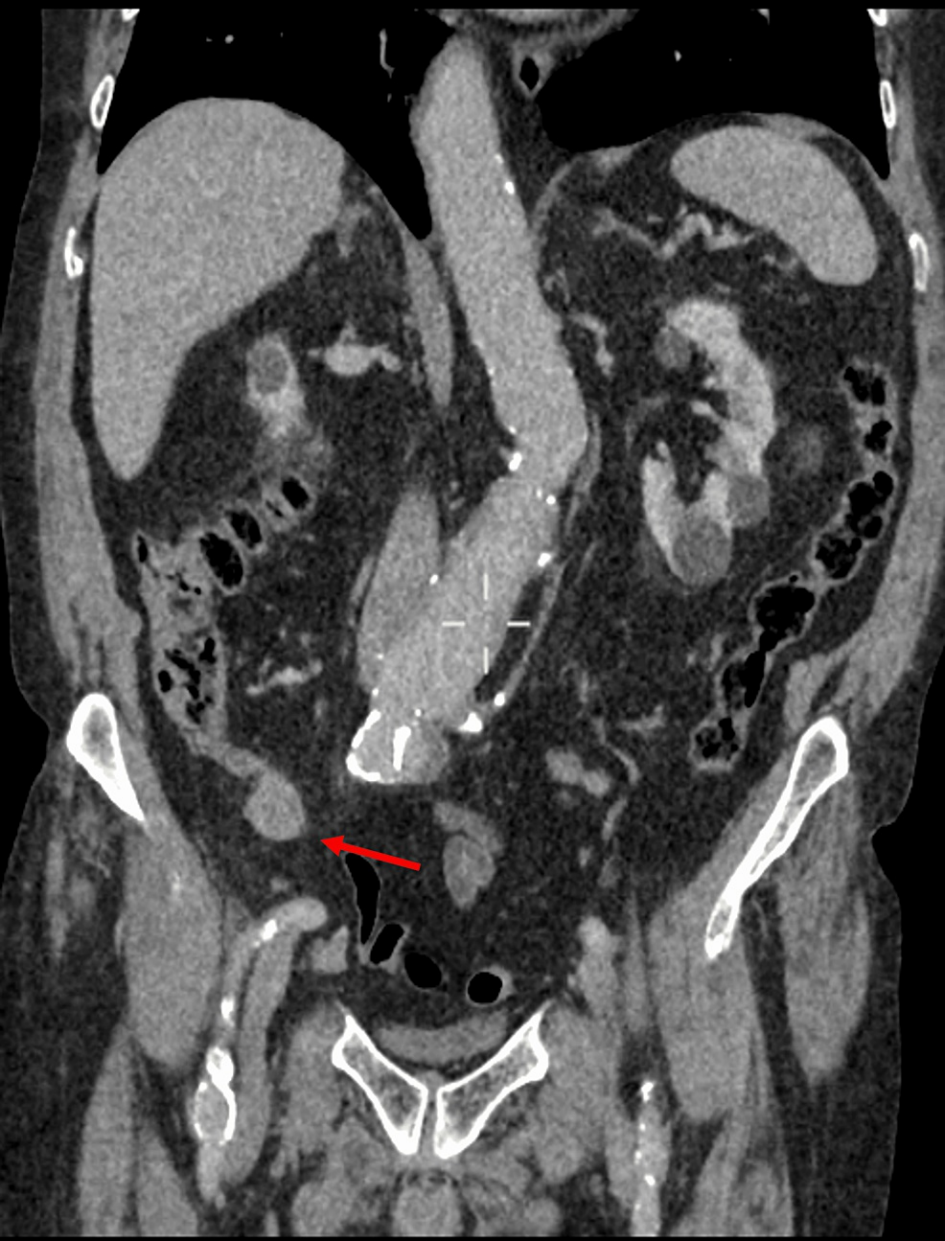
CT of the abdomen and pelvis - coronal section. The coronal cross-sectional CT image of this patient showing a fusiform appendiceal mass (red arrow) with no evidence of an inflammatory process or appendiceal obstruction.

**Figure 2:**
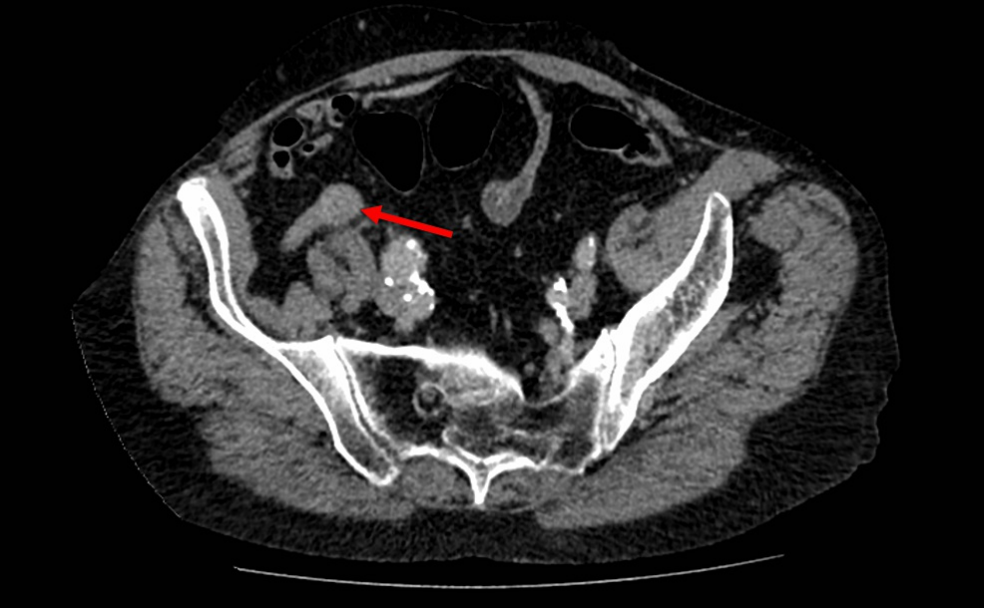
CT of the abdomen and pelvis - transverse section. A transverse cross-sectional CT image of this patient showing a fusiform appendiceal mass (red arrow). Other findings remain as described in Figure 1.

Given his relevant history, abdominal CT with contrast was performed (Figures 1, 2) which did not reveal a cause for his presentation. However, a dilated fusiform appendix measuring 2.2 cm × 2.2 cm was identified. There were no radiological signs of appendicitis, appendiceal faecoliths, or metastases. There was no focal lymphadenopathy. A subsequent CT of the thorax confirmed the absence of any pulmonary metastasis.

Treatment

**Figure 3:**
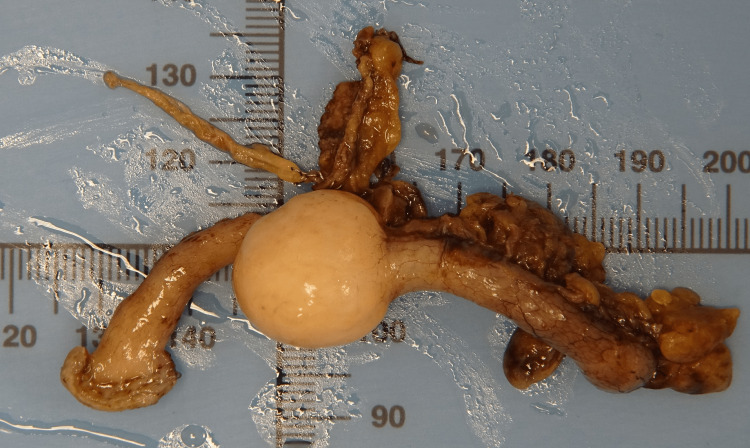
Surgical specimen. Gross surgical specimen of the appendix with the fusiform mass in the mid-appendix.

Following a discussion at a multidisciplinary team meeting, the patient underwent laparoscopic appendicectomy. Laparoscopy confirmed a distended bulbous appendix with no signs of inflammation or perforation. There was no evidence of intraperitoneal metastasis, mucin, or free fluid. There were small bowel adhesions related to his previous aneurysm repair. The delivered specimen is shown in Figure 3.

Outcomes and follow-up 

**Figure 4:**
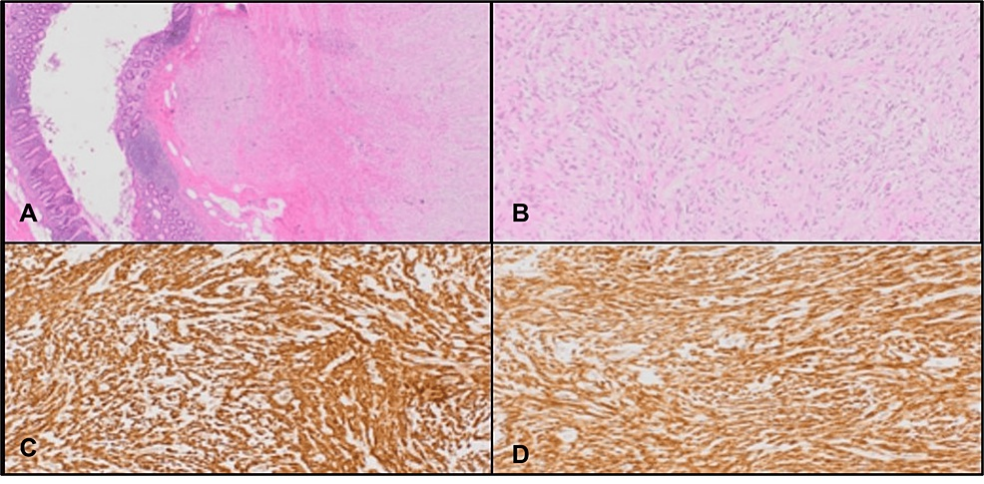
Magnified stained pathological sections. (A) A specimen section with hematoxylin and eosin (H&E) stain under ×20 magnification. Neoplastic well-circumscribed nodule centred within the muscularis propria undermining the normal appendiceal mucosa. (B) A specimen section with H&E stain under ×100 magnification. The tumour is composed of bland spindled cells arranged in fascicles. Nuclei are elongated with inconspicuous nucleoli and eosinophilic cytoplasm. There is no significant cytological atypia, mitosis (0/5 mm^2^), or necrosis. (C) A specimen section with CD34 stain under ×100 magnification. Neoplastic cells show diffuse membranous positivity with CD34. (D) A specimen section with CD117 stain under ×100 magnification. Neoplastic cells show diffuse cytoplasmic positivity with CD117 (c-kit).

Pathological examination (Figure 4) confirmed complete resection of a mitotically inactive spindle cell tumour measuring 1.5 cm in maximum dimension. CD34, CD117, and DOG1 testing were positive, confirming the diagnosis of GIST.

Ki-67 was <1%. Hence, mutational analysis was not performed given low-risk features. The case was reviewed by the pathology department in a tertiary centre where the diagnosis was confirmed.

This case was discussed by the Regional Sarcoma Tumour Board, where it was concluded that the tumour was very low-risk with no indication for adjuvant systemic anti-cancer therapy. No further follow-up was recommended.

At the two-month follow-up, the patient had recovered well without any postoperative complications. He was reviewed as an outpatient and discharged from follow-up. He has not since presented to secondary care with relevant symptoms at the time of writing.

## Discussion

Due to the rarity of this tumour type, current literature is limited to single case reports and small case series [3,4,6,7].

Given the limited body of evidence, the Royal College of Pathologists makes no recommendation on risk stratification for these tumours at this anatomic site [8]. However, the College of American Pathologists recommends that these tumours should be risk assessed in the same way as jejunal/ileal GISTs [9], which is also supported by the existing literature [4,5].

Identifying our case as low-risk is also supported by the previously mentioned study conducted by Hu et al. [3]. This study of rare GIST cases, including appendiceal GISTs, aimed to determine whether size and mitotic activity might predict risk, suggesting that small tumours with low mitotic activity were very unlikely to progress. Furthermore, most appendiceal GISTs fall into this category and can be managed curatively by appendicectomy [3].

Our case is similar to the majority of reported cases, being mitotically inactive and low risk based on current classification systems in part due to the low tumour sizes when these lesions are detected. There have been previous summaries of reported single case reports in the literature. Chun and Lim found that there was a possible predilection for men in the reported cases in the literature. Regarding clinical presentation, they identified that the two ways in which these cases present were either as a suspected acute appendicitis or an incidental finding during surgery for other indications or at autopsy, similar to our case where this was an incidental finding on cross-sectional imaging for symptoms likely unrelated to tumour [6]. They reported that most cases were managed with a simple appendicectomy, as with our case. Chun and Lim also found that histopathological examination of these tumours across the series of reported cases revealed that the majority had a low mitotic count and therefore likely low biological aggressiveness. However, these lesions are not universally low-risk. They also identified one case in the literature with a reported mitotic count of 9 mitoses/high-power field (hpf) [6,10]. In such cases, where there is evidence of advanced or aggressive disease features, tyrosine kinase receptor inhibitors have been used to improve outcomes [10,11]. In the single case with a high mitotic count, the patient was treated neoadjuvantly with imatinib [10]. The patient subsequently presented unwell with an acute abdomen necessitating an emergency exploratory laparotomy where a localised perforated appendiceal GIST adherent to an adjacent small bowel loop was found [10]. The patient underwent an en-bloc resection of this [10]. There is also a case report of a large-size appendiceal GIST associated with peritoneal metastasis [11]. In this case, the only reported instance of appendiceal GIST with distant metastases, a course of neoadjuvant oral imatinib led to a marked reduction in tumour burden, thus improving resectability. In this case, the tumour was larger with a higher mitotic index [11]. Our case, however, was in keeping with the majority of other reported cases; therefore, given the low-risk features in our case, mutational analysis and consideration of neoadjuvant therapy were not required.

In a large contemporaneous multi-centre study reviewing GIST diagnoses at rare anatomical sites within the institutions’ pathology archives, 27 appendiceal GISTs were identified across 47 centres [3]. In this cohort, there was predilection towards the female sex (66.7%) in contrast to a review of the reported cases performed by Chun and Lim of a male predilection [3,6]. The majority of cases were diagnosed incidentally. Similar to Chun and Lim’s findings, no cases in this cohort had a mitotic count >5/hpf [3]. Again the majority underwent an appendicectomy, although six patients in the cohort underwent more extensive surgery in the form of an appendicectomy and caecectomy or right hemicolectomy [3]. None of the cases suffered a recurrence at the end of follow-up [3]. While this study was able to validate the known utility of mitotic rate and tumour size in GISTs in the novel context of these tumours in the colon and oesophagus as rare sites, their case numbers for appendiceal GISTs meant that they could not replicate similar analyses for GISTs at this site again highlighting the difficulties dealing with the sparse clinical, pathological, and outcomes data associated with GISTs at this site [3].

## Conclusions

Appendiceal GISTs are rare entities, with the current literature suggesting these usually fall clinically within a lower-risk spectrum. However, risk classifications are currently extrapolated from GISTs at other sites due to a lack of appendix site-specific data. Similarly, data on its natural history, management, postoperative surveillance, and long-term outcomes remains limited in the reported literature.

Therefore, maintaining an international registry with mature follow-up data remains an important research and clinical need, with the potential for this data to guide, inform, and standardise clinical management of these tumours in the future. Management and follow-up decisions for these patients should continue to be multidisciplinary team-led; however, they should be supported by clinical guidelines, for which an increased body of data is required. In this case, we have highlighted the rarity of GISTs of the appendix and demonstrated a further example of these low-risk lesions, managed by excision only, based on a small but growing evidence base.
